# Protective effect of Salidroside on hypoxia‐related liver oxidative stress and inflammation via Nrf2 and JAK2/STAT3 signaling pathways

**DOI:** 10.1002/fsn3.2459

**Published:** 2021-07-10

**Authors:** Yanlei Xiong, Yueming Wang, Yanlian Xiong, Lianghong Teng

**Affiliations:** ^1^ Department of Pathology Xuanwu Hospital Capital Medical University Beijing China; ^2^ Department of Pathophysiology Institute of Basic Medical Sciences Chinese Academy of Medical Sciences (CAMS) School of Basic Medicine Peking Union Medical College (PUMC) Beijing China; ^3^ Department of anatomy School of Basic Medicine Binzhou Medical University Yantai China

**Keywords:** hypoxia, inflammation, liver, oxidative stress, salidroside

## Abstract

High‐altitude hypoxia‐induced oxidative stress and inflammation played an essential role in the incidence and development of liver injury. Salidroside (Sal), a phenylpropanoid glycoside extracted from the plant Rhodiola rosea, has recently demonstrated antioxidant, anti‐inflammatory, and antihypoxia properties. Herein, we hypothesized that salidroside may alleviate hypoxia‐induced liver injury via antioxidant and antiinflammatory‐related pathways. A high‐altitude hypoxia animal model was established using hypobaric chamber. Male *SD* rats were randomly divided into the control group, hypoxia group, control +Sal group, and hypoxia +Sal group. Salidroside treatment significantly inhibited hypoxia‐induced increases of serum and hepatic pro‐inflammatory cytokines release, hepatic ROS production and MDA contents; attenuated hypoxia‐induced decrease of hepatic SOD, CAT, and GSH‐Px activities. Furthermore, salidroside treatment also potentiated the activation of Nrf2‐mediated anti‐oxidant pathway, as indicated by upregulation of n‐Nrf2 and its downstream HO‐1 and NQO‐1. In vitro study found that blocking the Nrf2 pathway using specific inhibitor ML385 significantly reversed the protective effect of salidroside on hypoxia‐induced liver oxidative stress. In addition, salidroside treatment significantly inhibited hepatic pro‐inflammatory cytokines release via JAK2/STAT3‐mediated pathway. Taken together, our findings suggested that salidroside protected against hypoxia‐induced hepatic oxidative stress and inflammation via Nrf2 and JAK2/STAT3 signaling pathways.

## Graphical abstract

Protective mechanisms of salidroside against hypoxia‐induced liver injury. Salidroside protected liver from hypoxia‐induced oxidative stress via the Nrf2 signaling pathway. The anti‐inflammatory effect of salidroside was dependent on the inhibition of the JAK2/STAT3 pathway combining with the activation of the Nrf2 pathway.

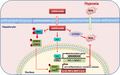

## 1 INTRODUCTION

Liver is considered as the largest metabolic organ and functions as the center of numerous metabolic and physiological processes, which plays a pivotal role in high‐altitude adaptation (Nath & Szabo, 2012). Liver function disorder may induce maladaption to high altitude and increase the incidence of acute mountain sickness (AMS) (Luks & Swenson, 2015). Although the molecular mechanisms underlying high‐altitude hypoxia‐induced liver injury remain widely unclear, both oxidative stress and inflammation response are considered as prominent factors involved in the pathogenesis of liver disorder under hypoxia.

Reactive oxygen species (ROS) are highly reactive species primarily generated in the mitochondria and in the endoplasmic reticulum of hepatocytes. (Prieto & Monsalve, 2017). Under normal conditions, ROS production can be effectively ameliorated via various antioxidant enzymes, such as superoxide dismutase (SOD), catalase (CAT), and glutathione peroxidase (GSH‐Px). However, oxidative stress is generated due to an imbalance between ROS production and antioxidant enzymes under stressful conditions (Cichoz‐Lach & Michalak, 2014). Hypoxia is considered as one of the most important contributors of oxidative stress, which plays an initiating role in the development of liver injury (Garnacho‐Castano et al., 2016; Sundaram et al., 2016). In addition, hypoxia exposure could also increase pro‐inflammatory cytokines release (Wang et al., 2016) and induce inflammatory response in the liver (Hernandez et al., 2020; Kang et al., 2017), which further aggravate liver dysfunction. Herein, potential therapeutic strategies targeting oxidative stress and inflammatory response showed promise to ameliorate the advance of liver disease (Ji et al., 2019; Musso et al., 2016).

Salidroside is the main ingredient of Rhodiola which has been reported for its anti‐apoptosis (Xiong et al., 2020), anti‐inflammatory (Pu et al., 2020), and antioxidant properties (Yang et al., 2016). Convincing evidence has emphasized its protective effects on the nervous and cardiovascular systems and inflammatory diseases, which function as a potent antioxidant and anti‐inflammatory response compound (Li et al., 2017; Liu et al., 2020). Recently, salidroside was reported to protect against furan‐induced hepatic injury by alleviating oxidative stress and systemic low‐grade inflammation (Yuan et al., 2019).

Oxidative stress combined with inflammatory response may accelerate hypoxia‐induced liver disorder. Although previous studies have demonstrated that salidroside possesses hepatoprotective effects in the liver with nonalcoholic steatohepatitis (Zheng et al., 2018) and CCl4‐induced liver injury (Lin et al., 2019) via ameliorate oxidative stress and inflammatory response. The protective effects of salidroside on high‐altitude hypoxia triggered liver injury and its potential molecular mechanism remain to be explored. Herein, the aim of this study is to elucidate the protective effect of salidroside on hypoxia‐induced liver oxidative stress and inflammatory reaction.

## 2 MATERIALS AND METHODS

### 2.1 Animals care

Adult male Sprague Dawley rats (280–330 g) was obtained from Weitong lihua Laboratory Animal Limited Company. The rats were housed at room temperature (20–22°C) and in a 12 hr–12 hr light–dark cycle with free access to food and water and adapted to the condition above for 1 week before the experiment. All experiments were conducted in accordance with the laboratory animal care guidelines published by the US National Institutes of Health (NIH publication no. 85‐23, revised 1996). All protocols concerning animal use were approved by the Institutional Animal Care and Use Committee of Institute of Basic Medical Sciences, Peking Union Medical College and Capital Medical University.

### 2.2 Hypoxic challenge

Adult male *SD* rats were randomly divided into four groups (*n* = 6/each group): (1) Control group, (2) Hypoxia group, (3) Control +Sal (20 mg/kg body weight) and (4) Hypoxia +Sal (20 mg/kg body weight). The rats in the Hypoxia group and Hypoxia +Sal group were raised in a hypobaric chamber (Guizhou Fenglei Air Ordnance Co., Ltd.) and subjected to hypoxia mimicking an altitude of 5,500 m for 10 days. The chamber was opened daily for 30 min to clean and replenish food and water. Meanwhile, the rats in the Control +Sal and Hypoxia +Sal groups were administered salidroside (dissolved in 0.9% saline) at doses of 20 mg kg^−1^ day^−1^ body weight by intraperitoneal injection before the hypoxic challenge for a total of 10 days. Salidroside (purity >98%) was purchased from the National Institute for Food and Drug Control (Beijing, China). All the rats were sacrificed by decapitation and serum was obtained by centrifugation and stored at −80°C. The liver tissue was quickly collected and weighed, frozen in liquid nitrogen, and stored at −80°C.

### 2.3 Culture of human hepatic cell line L02 cells

The human hepatic cell line L02 cells were obtained from the cell bank of Type Culture Collection of the Chinese Academy of Sciences (Shanghai, China). L02 cells were cultured in a medium mixed with DMEM, 10% (v/v) FBS, and 100 units/ml penicillin (in 5% CO_2_, 37°C). L02 cells were preincubated in phenol red‐free and serum‐free DMEM in an atmosphere of 5% CO_2_ at 37°C for 1 hr before the treatment. For hypoxia exposure, cells were incubated with the serum‐free medium and placed in an airtight humidified chamber with 37°C, 5% CO_2_, and 95% N_2_. The corresponding normoxia control cells were cultured in a humidified incubator with 37°C, 5% CO_2_, and 21% O_2_. ML385 (inhibitor of Nrf2) and AG490 (inhibitor of JAK2) were purchased from Sigma‐Aldrich. ML385 (10 μmol/L), AG490 (10 μmol/L) and salidroside (10 μmol/L) were used to treat L02 cells.

### 2.4 Histopathological observation of liver tissues

The liver tissues of rat were fixed in 4% paraformaldehyde overnight, followed by embedment in paraffin and longitudinal slicing, with 4 μm thick sections obtained and stained with hematoxylin and eosin (H&E). Immunohistochemistry (IHC) staining was conducted to examine the expression of Nrf2 in the liver. Briefly, the sections were deparaffinized and rehydrated, then incubated with a hydrogen peroxide block for 15 min. The sections were incubated with Nrf2 primary antibody (1:500, Cell Signaling Technology) overnight at 4°C followed by incubation in biotinylated secondary antibody for 1 hr at room temperature. After incubation with DAB chromogen, the sections were ultimately counterstained with hematoxylin. The stained slides were examined for histomorphological analyses at 400× magnification using optical microscope.

### 2.5 Western blotting and densitometry analyses

Homogenized rat liver was lysed in 200 μl RIPA lysis buffer (Beyotime, P0013B) with 1% phenylmethyl sulfonylfluoride and 4% complete protease inhibitor cocktail mix (Roche, Mannheim, Germany). Extracts were centrifuged at 14,000 g for 15 min at 4°C. Eighty micrograms of total protein were used for sodium dodecyl sulfate‐polyacrylamide gel electrophoresis, followed by transferring blotting to nitrocellulose membrane (Millipore Corp., Billerica, MA, USA). Membranes were then blocked with 5% nonfat dried milk in PBS for 1 hr with gentle shaking. Membranes were incubated first with anti‐Janus kinase 2(JAK2), anti‐signal transducer and activator of transcription 3 (STAT3) and their phosphorylated species, anti‐cytochrome P450 2E1(CYP2E1), anti‐Nuclear factor erythroid 2 related factor 2 (Nrf2), anti‐heme oxygenase‐1(HO‐1), anti‐NAD(P)H: quinone oxidoreductase 1 (NQO‐1), and anti‐β‐actin antibody [Cell Signaling Technology] were incubated overnight at 4°C, in 1% BSA in PBS overnight at 4°C with shaking, washed and incubated with secondary antibodies for 2 hr at room temperature. Finally, the samples were visualized by enhanced chemi‐luminescence. After scanning, band density was analyzed using Image J 1.33 software (National Institutes of Health, Bethesda, MD, USA).

### 2.6 Serum measurements

The concentrations of pro‐inflammatory biomarkers, namely interleukin‐1β (IL‐1β), tumor necrosis factor‐α (TNF‐α), interleukin‐6 (IL‐6), and monocyte chemoattractant protein‐1(MCP‐1) were determined by an enzyme‐linked immunosorbent assay (ELISA) according to the manufacturer's instructions (IBL International GmbH). A standard curve was used to converting the OD reads and calculated the amount of IL‐1β, IL‐6, TNF‐α, and MCP‐1 in the samples.

### 2.7 Determination of ROS production, MDA and AGEs activity in the liver

The weighed liver tissue samples and collected L02 cells were homogenized in PBS to prepare 10% homogenate, then the supernatant was collected after centrifugation at 4°C, 10,000 *g* for 10 min. The ROS production, MDA and AGEs in the supernatant of liver homogenate were determined according to the requirements of the manufacturer's protocol in reagent kits (Nanjing Jiancheng Bioengineering Institute, China). The protein concentration in the liver and L02 cells was determined using BCA protein assay reagent kit. The values of ROS production, MDA, and AGEs were normalized to liver tissue protein concentration, respectively.

### 2.8 Determination of antioxidant status

The GSH‐Px, CAT, and SOD activity in liver tissues were detected by colorimetric analysis according to the manufacturer's protocol were determined according to the requirements of the instructions provided in reagent kits (Xiao et al., 2018).

### 2.9 Reverse‐transcription PCR and quantitative real‐time PCR

Total RNA was prepared from liver tissues and L02 cells with trizol reagent (Invitrogen) and the cDNA was synthesized using TransScript TM First‐Strand cDNA Synthesis Super‐Mix (TransGen Biotech, AT301). Quantitative real‐time PCR was performed using the SYBR^®^Pre‐mix Ex TaqTMkit (Takara, RR420A) and analyzed in a step‐one plus RT‐PCR system (life science, Applied Biosystems). The primer sequences of rat IL‐1β, TNF‐α, and IL‐6 were referenced (Song et al., 2016). The primer sequences of rat MCP‐1 were 5′‐GGCCTGTTGTTCACAGTTGCT‐3′ (sense) and 5′‐TCTCACTTGGTTCTGGTC CAGT‐3′ (antisense); The primer sequences of rat β‐actin were 5′‐CGTTGACATCCGTAAAGACC‐3′ (sense) and 5′‐GCTAGGAGCCAGGGCAGTA‐3′ (antisense). The primer sequences of human IL‐1β, TNF‐α, and IL‐6 were referenced (Sun et al., 2000). The primer sequences of human MCP‐1 were 5′‐CAGATGCAATCAATGCCCCAGT‐3′ (sense) and 5′‐ATAAAA CAGGGTGTCTGGGGAAAGC‐3′ (antisense). The primer sequences of human β‐actin were 5′‐CGTACCACTGGCATCGTGAT‐3′ (sense) and 5′‐GTGTTGGCGTACAGGTCTTTG ‐3′ (antisense). Relative mRNA expression of genes was calculated using 2^−∆∆^CT method.

### 2.10 Statistical analysis

The data are presented as mean ± standard error (*SE*). Statistical significance is determined by one‐way analysis of variance (ANOVA) with multiple comparisons or nonparametric test. A *p*‐value < .05 was considered statistically significant (SPSS 18.0 software).

## 3 RESULTS

### 3.1 Salidroside attenuated hypoxia‐induced liver oxidative stress

**FIGURE 1:**
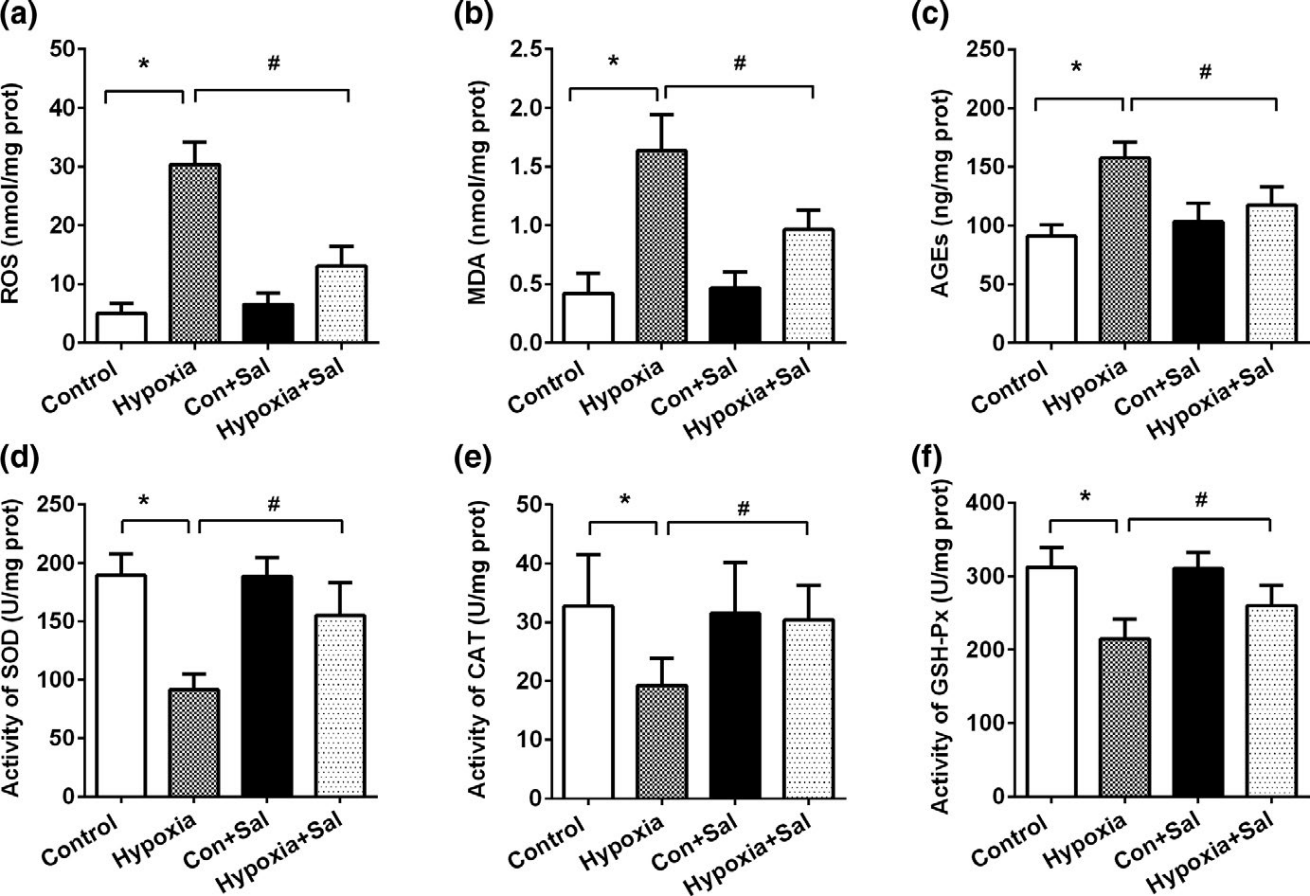
Salidroside attenuated hypoxia‐induced liver oxidative stress. Hepatic levels of (a) ROS, (b) MDA, (c) AGEs, (d) SOD activity, (e) CAT activity, and (f) GSH‐Px activity. Data are shown as mean ± *SE*, *p < .05, **p < .01, (Control group versus Hypoxia group, *n* = 6/group). #*p* < .05, ##*p* < .01 (Hypoxia group versus Hypoxia +Sal group, *n* = 6/group)

Hypoxia exposure could accelerate ROS production and evoke oxidative stress, gradually leading to liver dysfunction. Levels of oxidative stress biomarkers (ROS, MDAs, and AGEs) significantly increased in the hypoxia group (*p* < .05), which were effectively ameliorated by salidroside treatment (*p* < .05) (Figure 1a–c). Levels of hepatic antioxidant enzymes, namely SOD, CAT, and GSH‐Px activities, showed significant decline in hypoxia group compared with control group (*p* < .05), due to their rapid clearance when coping with oxidative stress. Salidroside treatment significantly attenuated hypoxia‐induced decline of SOD, CAT, and GSH‐Px activities compared with the hypoxia group (*p* < .05) (Figure 1d–f).

### 3.2 Salidroside ameliorated hypoxia‐induced liver inflammation

**FIGURE 2:**
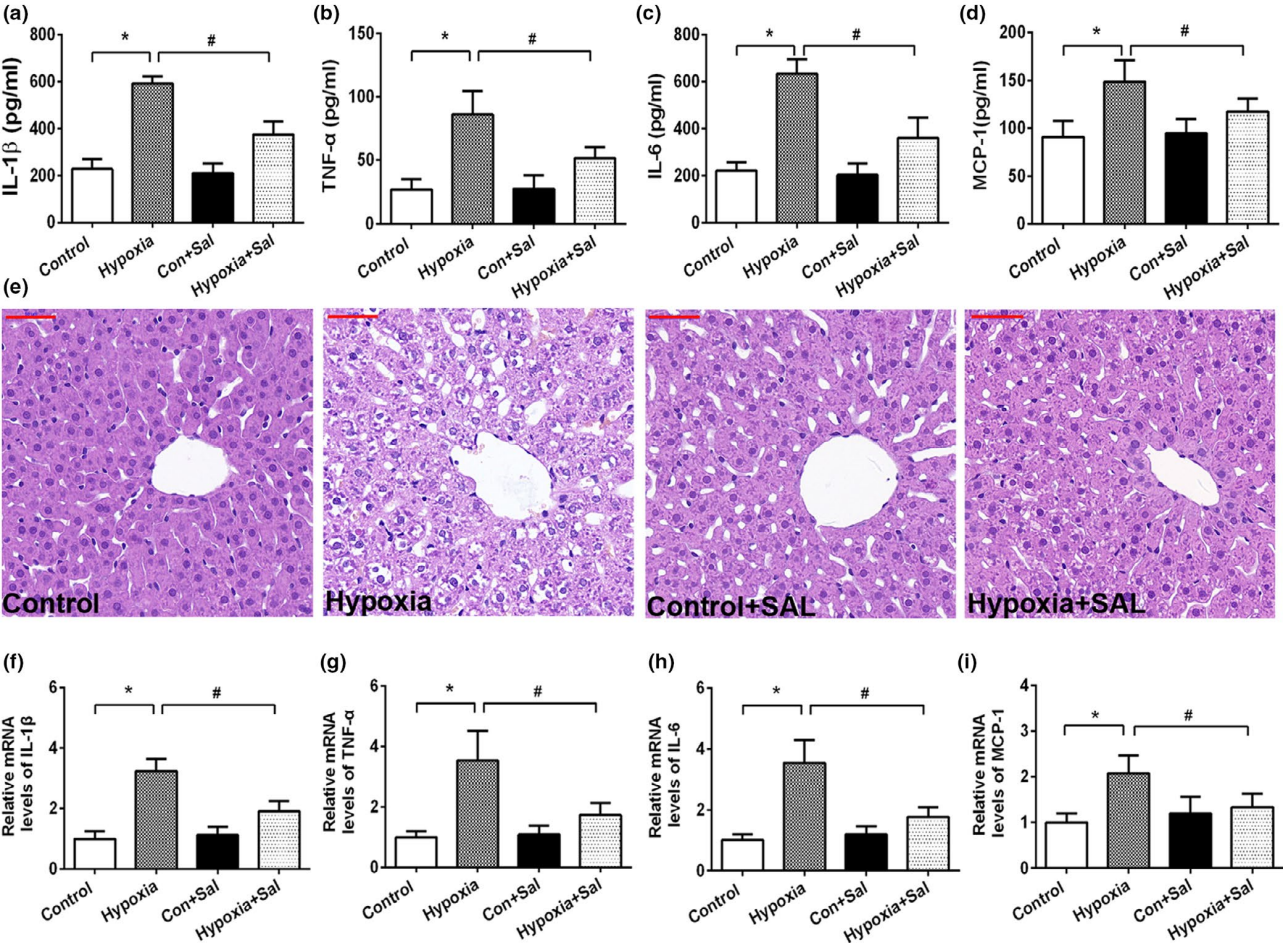
Salidroside ameliorated hypoxia‐induced liver inflammation. Serum levels of (a) IL‐1β, (b) TNF‐α, (c) IL‐6, and (d) MCP‐1; (e) Representative images of HE‐stained liver sections (magnification 400×, scale bar = 50 μm); mRNA expression levels of (f) IL‐1β, (g) TNF‐α, (h) IL‐6, and (i) MCP‐1 in liver tissue. Data are shown as mean ± *SE*, *p < .05, **p < .01, (Control group versus Hypoxia group, *n* = 6/group). ^#^ *p* < .05, ^##^ *p* < .01, (Hypoxia group versus Hypoxia +Sal group, *n* = 6/group)

To explore the effect of salidroside on pro‐inflammatory cytokines, serum levels of IL‐1β, TNF‐α, IL‐6, and MCP‐1 were measured in rats treated with or without salidroside after hypoxia. Compared to the hypoxia group, the augment of pro‐inflammatory cytokines including IL‐1β, TNF‐α, IL‐6, and MCP‐1, was significantly suppressed in hypoxia +Sal group rats (Figure 2a–d). In addition, representative histopathological changes of the liver tissues are shown in Figure 2e. The control group exhibited a normal lobular architecture and clear hepatic cords, whereas the basic architecture of hepatocytes was disappeared in hypoxia group, accompanied with distorted hepatic cords, cellular swelling, and hyperemia. Treatment of salidroside significantly ameliorated pathohistological alterations in the liver of hypoxia rats. Meanwhile, a supplement with salidroside also restrained the increased mRNA levels of IL‐1β, TNF‐α, IL‐6, and MCP‐1 in rat liver upon hypoxia (Figure 2f–i). The data above suggested a protective effect of salidroside against hypoxia‐induced inflammation response.

### 3.3 Salidroside protected liver from hypoxia‐induced oxidative stress via the Nrf2 signaling pathway

**FIGURE 3:**
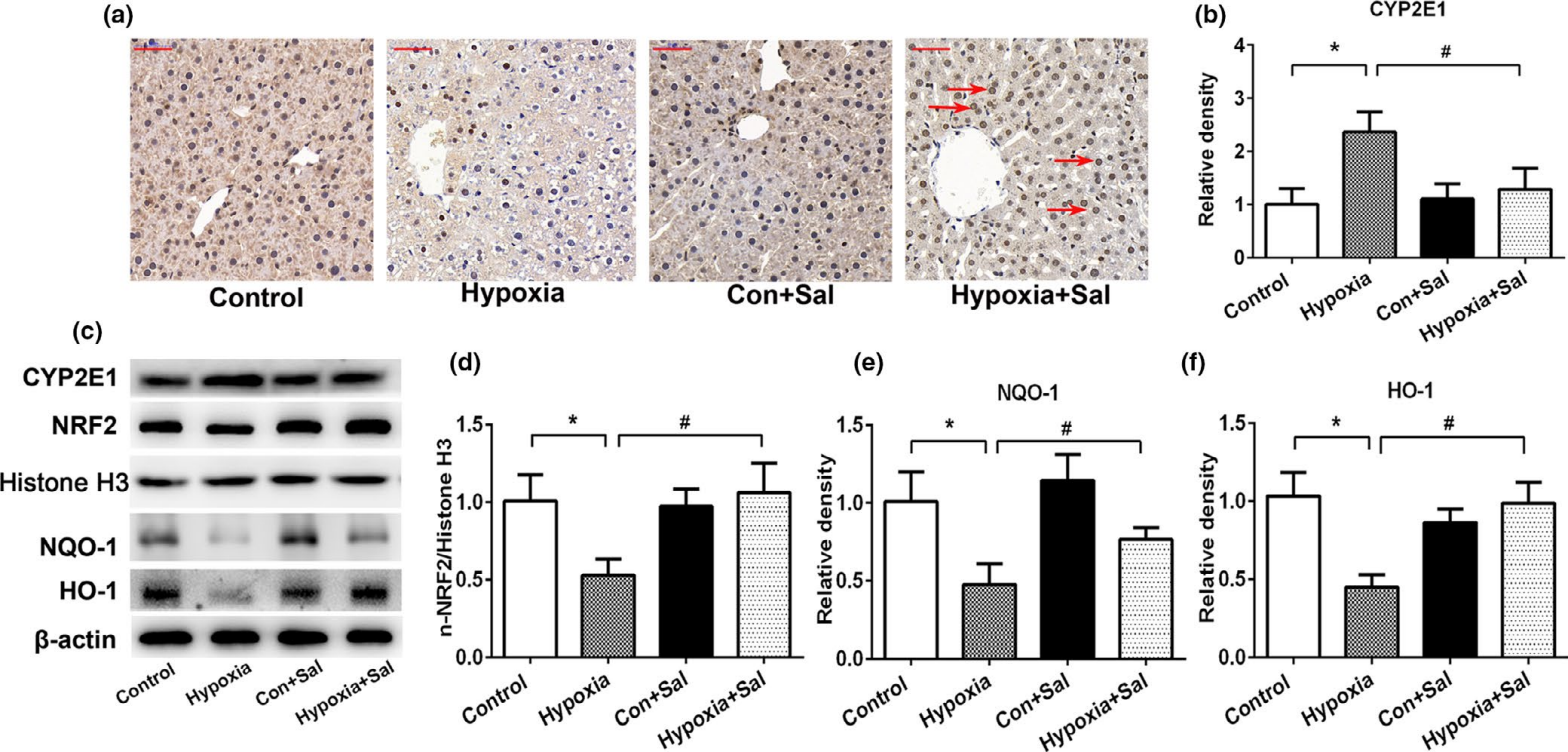
Salidroside protected liver from hypoxia‐induced oxidative stress via Nrf2 signaling pathway. (a) IHC staining of Nrf2 in the liver sections. Red arrows indicate the expression of Nrf2 translocated into the nucleus in the liver. (magnification 400×, scale bar = 50 μm) (b) Relative CYP2E1 protein expression levels; (c) The protein expressions of CYP2E1, Nrf2, NQO‐1, and β‐actin. (d) Relative Nrf2 protein expression levels; (e) Relative NQO‐1 protein expression levels; (f) Relative HO‐1 protein expression levels. Data are shown as the mean ± *SE*, *p < .05, **p < .01, and ***p < .001 (control versus 5.5 km hypoxia, *n* = 6/group). #*p* < .05, ##*p* < .01 (5.5 km hypoxia versus 5.5 km hypoxia +Sal, *n* = 6/group)

To further explore the potential antioxidant mechanisms of salidroside on hypoxia‐induced liver oxidative stress, the expressions of CYP2E1 and Nrf2‐related pathways in liver tissues were conducted. Salidroside treatment significantly inhibited the hypoxia‐induced upregulation of CYP2E 1 expression (Figure 3b). Under normal conditions, Nrf2 is localized in the cytoplasm, while under conditions of oxidative stress, Nrf2 may translocate into the nucleus and regulate the expression of downstream antioxidant genes HO‐1 and NQO‐1 (Zhang et al., 2017). As shown in Figure 3a, the salidroside‐treated group altered the hypoxia‐inhibited Nrf2 expression in the liver. Nrf2 translocated into the nucleus in hypoxia +Sal group (red arrow), indicating activation of the Nrf2‐related pathway by salidroside. The nuclear expressions of Nrf2 significantly decreased in the hypoxia group (Figure 3d). Meanwhile, levels of Nrf2 downstream target gene NQO1, HO‐1, also significantly decreased (Figure 3e, f). Treatment with salidroside significantly attenuated hypoxia‐induced decrease of n‐Nrf2, NQO‐1, and HO‐1. These data suggest that salidroside ameliorate liver injury from hypoxia‐induced oxidative stress via inhibiting CYP2E1expression and activating the Nrf2‐related pathway.

**FIGURE 4:**
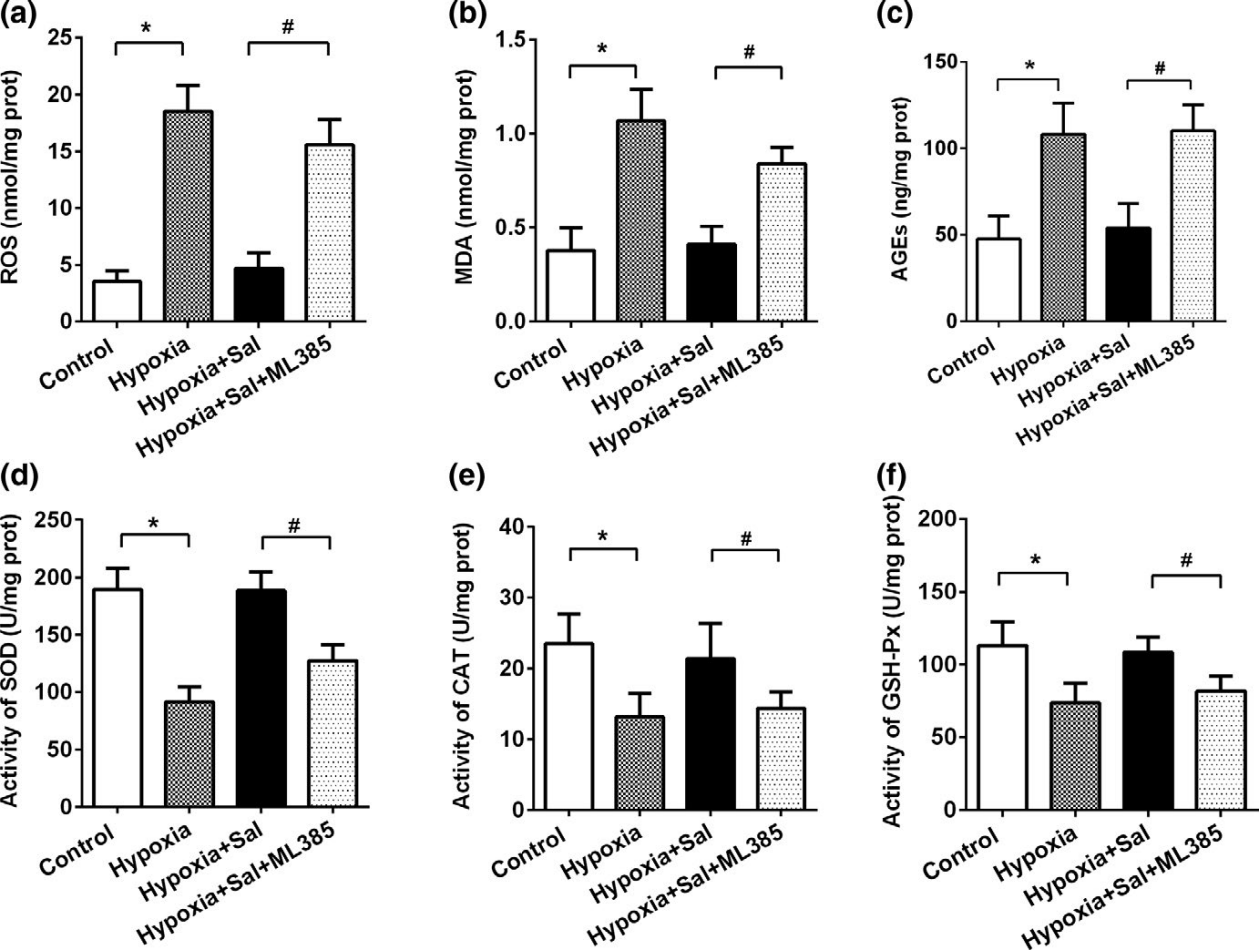
Salidroside protected liver from hypoxia‐induced oxidative stress via Nrf2 signaling pathway. The human hepatic cell line L02 cells were pretreated with or without Salidroside or ML385 for 6 hr, then followed by hypoxia. Cellular levels of (a) ROS, (b) MDA, (c) AGEs, (d) SOD activity, (e) CAT activity, and (f) GSH‐Px activity. Data are shown as mean ± *SE*, *p < .05, **p < .01, (Control group versus Hypoxia group). #*p* < .05, ##*p* < .01 (Hypoxia +Sal group versus Hypoxia +Sal + ML385 group)

To further explore a possible link between salidroside and the Nrf2 signaling pathway, we examined the oxidative stress biomarkers and antioxidant enzymes in L02 cells treated with or without salidroside and ML385 (inhibitor of Nrf2). As shown in Figure 4, Levels of oxidative stress biomarkers (ROS, MDAs, and AGEs) significantly increased in the hypoxia group as compared with the control group, which was significantly attenuated by salidroside administration. Hypoxia exposure significantly decreased SOD, CAT, and GSH‐Px activity, which was attenuated by salidroside administration. However, treatment with the Nrf2 inhibitor ML385 significantly abolished these effects conferred by salidroside. These data indicated that salidroside suppressed hypoxia‐induced liver oxidative stress via the Nrf2‐related pathway.

### 3.4 Salidroside intervened with the JAK2/STAT3 pathway to ameliorate liver inflammation

**FIGURE 5:**
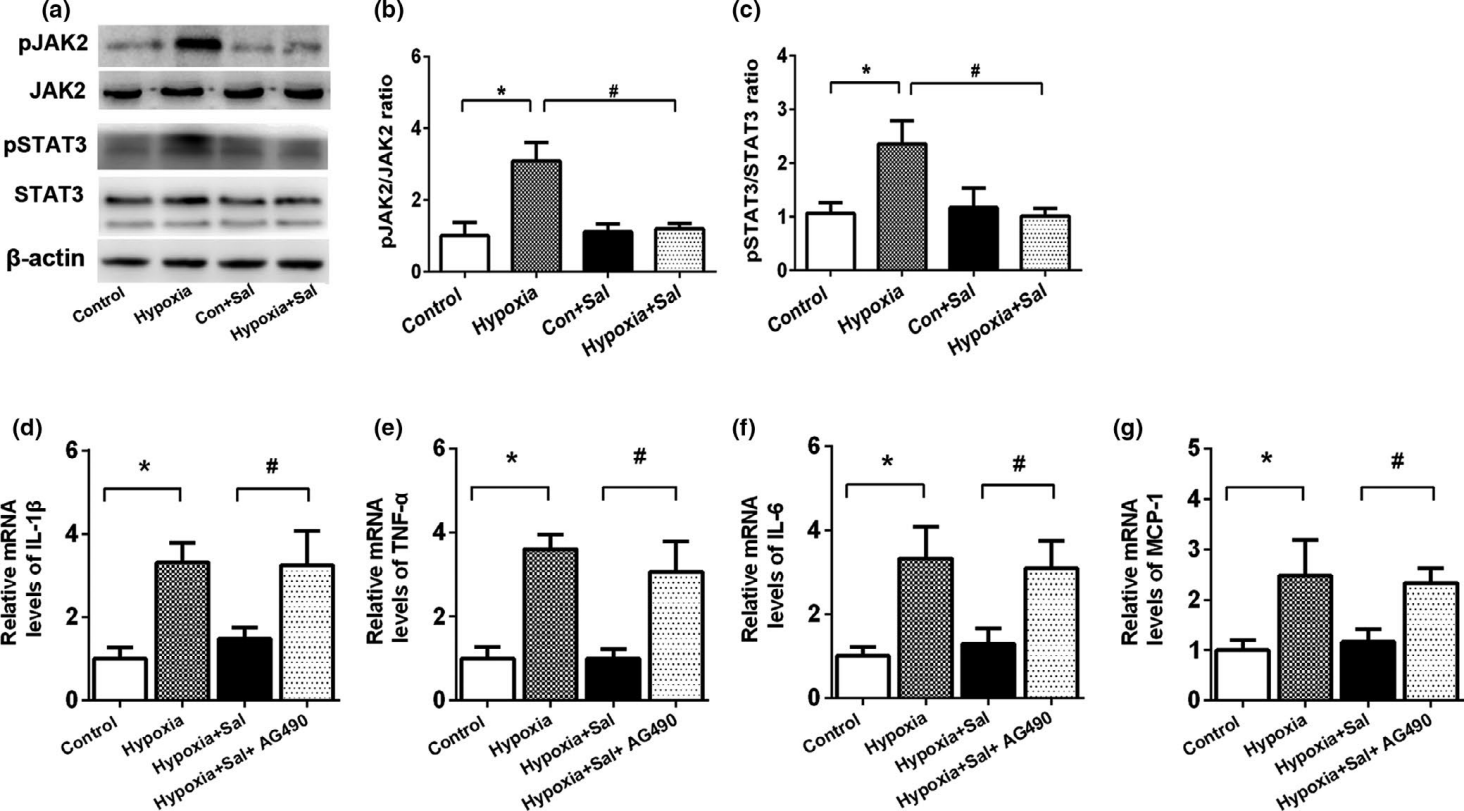
Salidroside inhibited JAK2/STAT3 signaling pathway to ameliorate hypoxia‐induced liver inflammation. (a) The protein expressions of p‐JAK2, JAK2, p‐STAT3, STAT3, and β‐actin in rat liver tissues; (b) p‐JAK2/JAK2 ratio; (c) p‐STAT3/ STAT3 ratio; Data are shown as the mean ± *SE*, *p < .05, **p < .01, and ***p < .001 (control versus 5.5 km hypoxia, *n* = 6/group). #*p* < .05, ##*p* < .01(5.5 km hypoxia versus 5.5 km hypoxia +Sal, *n* = 6/group). The human hepatic cell line L02 cells were pretreated with or without salidroside or AG490 for 6 hr, then followed by hypoxia. mRNA expression levels of (d) IL‐1β, (e) TNF‐α, (f) IL‐6, and (g) MCP‐1. *p < .05, **p < .01, (Control group versus Hypoxia group). #*p* < .05, ##*p* < .01 (Hypoxia +Sal group versus Hypoxia +Sal + AG490 group)

JAK2‐STAT3 is a key transcription factor involved in inflammatory cytokine release in liver disease (Gao, 2005). We explored the effect of salidroside treatment on JAK2‐STAT3 signaling. Protein levels of p‐JAK2 and p‐STAT3 were analyzed in the liver of the four groups of rats. As shown in Figure 5a–c, the ratios p‐JAK2/JAK2 and p‐STAT3/STAT3 significantly increased in the hypoxia group as compared with the control group, while salidroside treatment significantly inhibited p‐JAK2 and p‐STAT3 levels in hypoxia rats.

To further explore a possible link between salidroside and JAK2‐STAT3‐mediated pro‐inflammatory cytokines production, we examined the mRNA levels of IL‐1β, TNF‐α, IL‐6, and MCP‐1 in L02 cells treated with or without salidroside and AG490 (inhibitor of JAK2). Interestingly, the inhibitory effect of salidroside on hypoxia‐induced pro‐inflammatory cytokines release was almost completely abolished by the JAK2 inhibitor AG490 (Figure 5d–g). These data indicate that salidroside treatment could suppress hypoxia‐induced inflammation reaction by regulating the JAK2/STAT3 pathway in the liver.

## 4 DISCUSSION

Liver function is crucial in high‐altitude adaptation and is likely attacked by ROS and inflammation. Our findings demonstrate that salidroside can protect the liver against hypoxia‐induced oxidative stress and inflammation, as evidenced by inhibiting ROS production and pro‐inflammatory cytokines release. The beneficial effect of salidroside on hypoxia‐induced liver injury rely on activating the Nrf2‐related antioxidant pathway and restraining the JAK2/STAT3‐mediated inflammatory reaction.

Hypoxia exposure could accelerate the production of ROS and evoke oxidative stress (Irarrazaval et al., 2017). As reported by Li et al., mice exposed to hypobaric hypoxia markedly inhibited the activity of SOD and GSH and increased the levels of MDA and oxidized glutathione in the serum and liver (Li et al., 2020). Furthermore, oxidative stress could induce apoptosis and necrosis in hepatocytes, which finally lead to liver dysfunction (Li et al., 2015). In this study, we found that salidroside treatment significantly downregulated ROS, MDA, and AGEs contents, while significantly upregulated the activities of the antioxidative enzymes in the hypoxia group rat liver. These data indicated a potential protective role of salidroside by counteracting oxidative stress induced by hypoxia.

CYP2E1 is one of the main members of CYP450 family, mainly present in liver microsomes and plays a vital role in ROS production and liver injury (Lee et al., 2020). Nrf2 is considered as a master regulator that controls the cellular redox state under harmful stresses (Bellezza et al., 2018). As previously described, salidroside exerts its antioxidant effect by modulating the Nrf2 signaling pathway (Cai et al., 2017; Zhu et al., 2016). In addition, salidroside was reported to suppress ROS production through Akt and Nrf2‐regulated genes HO‐1 and NQO‐1 (Zheng et al., 2014). We speculated that the antioxidant property of salidroside may be achieved via activation of the Nrf2‐related pathway. As expected, we observed that hypoxia exposure upregulated the expression of CYP2E1 and downregulated Nrf2, and its downstream target genes HO‐1, NQO1. We further demonstrated that salidroside treatment significantly suppressed CYP2E1‐mediated ROS generation and activated the Nrf2 antioxidant pathway, which exert therapeutic effects on hypoxia‐induced injury by restoring cellular ROS homeostasis. Furthermore, in vitro study found that blocking the Nrf2 pathway using the specific inhibitor ML385 subsequently abolished these effects conferred by salidroside.

It is well known that excessive pro‐inflammatory cytokines release is another crucial trigger of hepatocyte damage. Cytokine signal transduction is predominantly mediated through the JAK/STAT pathway in the process of inflammation. STAT3, a member of the STAT family, is an important transcription factor associated with cytokine release and liver inflammation (Li et al., 2018). Previous study suggested that salidroside could reduce LPS‐induced pro‐inflammatory cytokines production and attenuate acute lung injury by inhibiting the JAK2‐STAT3 signaling pathway (Qi et al., 2016). Consistent with their findings, our results also showed that salidroside could restrain the serum and hepatic pro‐inflammatory cytokines release, including IL‐1β, TNF‐α, MCP‐1, and IL‐6. Furthermore, hypoxia‐induced activation of JAK2/STAT3 was inhibited by salidroside treatment, which is possibly a potential mechanism to ameliorate hypoxia‐induced liver inflammation. Blocking the JAK2/STAT3 pathway using a specific inhibitor AG490 subsequently reversed the protective effect of salidroside on liver inflammation.

**FIGURE 6:**
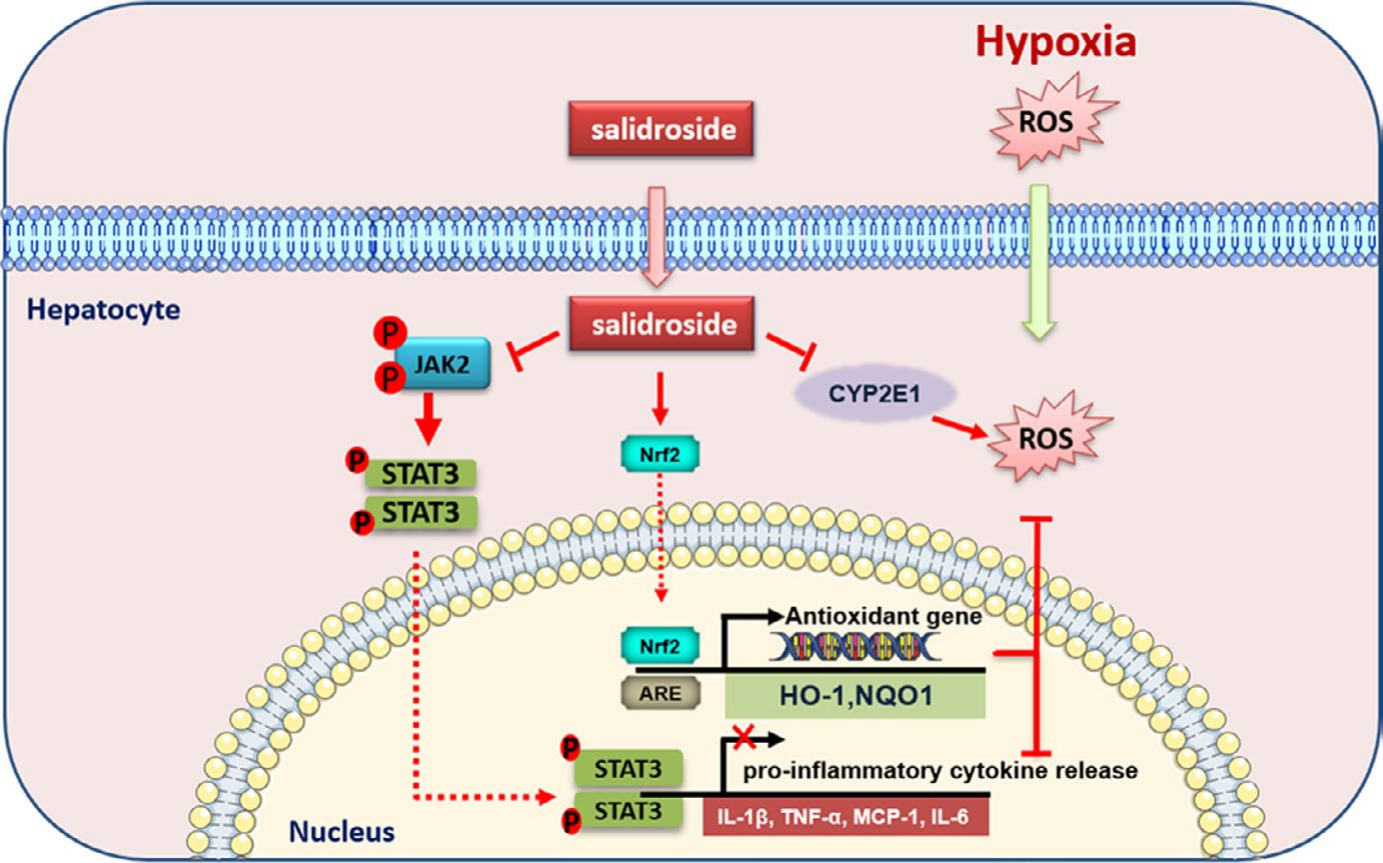
Protective mechanisms of salidroside against hypoxia‐induced liver injury. Salidroside protected liver from hypoxia‐induced oxidative stress via Nrf2 signaling pathway. The anti‐inflammatory effect of salidroside was dependent on inhibition of JAK2/STAT3 pathway combining with activation of Nrf2 pathway

Oxidative stress is closely correlated with inflammatory response, especially under hypoxia conditions (McGarry et al., 2018). High levels of ROS produced during oxidative stress stimulate the release of pro‐inflammatory mediators and increase inflammation, which may further aggravate liver injury. Moreover, activation of Nrf2 not only regulates oxidative stress response, but also contributes to the anti‐inflammatory process by regulating cytokines secretion (Ahmed et al., 2017). In our study, high‐altitude hypoxia not only induced hepatic oxidative stress, but also promoted pro‐inflammatory cytokines release synchronously. The oxidative stress combined with inflammation reaction jointly exacerbated pathological alterations in the liver under hypoxia exposure. As shown in Figure 6, the protective effects of salidroside on hypoxia‐induced liver injury mainly depending on the activation of the Nrf2‐mediated antioxidant pathway. While the anti‐inflammatory effect of salidroside was dependent on inhibition of the JAK2/STAT3 pathway combining with the activation of the Nrf2 pathway. It is also a common phenomenon that natural compounds may exert a protective effect on liver due to their antioxidant and anti‐inflammatory function. For instance, triptriolide could alleviate LPS‐induced oxidative stress and inflammation by regulating the Nrf2 and NF‐κB signaling pathways in the liver (Yang et al., 2018). Similar to our findings, G‐Rg2 and ‐Rh1 exerts a protective effect on liver function through inhibiting the TAK1 and STAT3‐mediated inflammatory activity and the Nrf2/ ARE‑mediated antioxidant signaling pathway (Nguyen et al., 2021).

## 5 CONCLUSIONS

In conclusion, our finding suggested that salidroside could ameliorate hypoxia‐induced liver oxidative stress and inflammation via Nrf2 and JAK2/STAT3 signaling pathways, which is likely to be a therapeutic candidate for the prevention and treatment of liver disorders at high altitudes.

## CONFLICT OF INTEREST

The authors declare no conflict of interests.

## AUTHOR CONTRIBUTIONS

**Yanlei Xiong:** Data curation (equal); Formal analysis (equal); Funding acquisition (equal); Investigation (equal); Methodology (equal). **Yueming Wang:** Formal analysis (equal); Methodology (equal). **Yanlian Xiong:** Data curation (equal); Funding acquisition (equal); Investigation (equal). **Lianghong Teng:** Conceptualization (equal); Investigation (equal).

## ETHICAL APPROVAL

Animal experiments were approved by the Institutional Animal Care and Use Committee of Institute of Basic Medical Sciences, Peking Union Medical College and Capital Medical University.

## Data Availability

The data that support the findings of this study are available from the corresponding author upon reasonable request.

## References

[fsn32459-bib-0001] Ahmed, S. M., Luo, L., Namani, A., Wang, X. J., & Tang, X. (2017). Nrf2 signaling pathway: Pivotal roles in inflammation. Biochimica Et Biophysica Acta (BBA) ‐ Molecular Basis of Disease, 1863(2), 585–597. 10.1016/j.bbadis.2016.11.005 27825853

[fsn32459-bib-0002] Bellezza, I., Giambanco, I., Minelli, A., & Donato, R. (2018). Nrf2‐Keap1 signaling in oxidative and reductive stress. Biochimica Et Biophysica Acta (BBA) ‐ Molecular Cell Research, 1865(5), 721–733. 10.1016/j.bbamcr.2018.02.010 29499228

[fsn32459-bib-0003] Cai, L., Li, Y., Zhang, Q., Sun, H., Yan, X., Hua, T., Zhu Q., Xu H., & Fu, H. (2017). Salidroside protects rat liver against ischemia/reperfusion injury by regulating the GSK‐3beta/Nrf2‐dependent antioxidant response and mitochondrial permeability transition. European Journal of Pharmacology, 806, 32–42. 10.1016/j.ejphar.2017.04.011 28411054

[fsn32459-bib-0004] Cichoz‐Lach, H., & Michalak, A. (2014). Oxidative stress as a crucial factor in liver diseases. World Journal of Gastroenterology, 20(25), 8082–8091. 10.3748/wjg.v20.i25.8082 25009380 PMC4081679

[fsn32459-bib-0005] Gao, B. (2005). Cytokines, STATs and liver disease. Cellular and Molecular Immunology, 2(2), 92–100. 16191414

[fsn32459-bib-0006] Garnacho‐Castano, M. V., Alva, N., Sanchez‐Nuno, S., Bardallo, R. G., Palomeque, J., & Carbonell, T. (2016). Hypothermia can reverse hepatic oxidative stress damage induced by hypoxia in rats. Journal of Physiology and Biochemistry, 72(4), 615–623. 10.1007/s13105-016-0500-x 27387890

[fsn32459-bib-0007] Hernandez, A., Geng, Y., Sepulveda, R., Solis, N., Torres, J., Arab, J. P., Barrera, F., Cabrera, D., Moshage, H., & Arrese, M. (2020). Chemical hypoxia induces pro‐inflammatory signals in fat‐laden hepatocytes and contributes to cellular crosstalk with Kupffer cells through extracellular vesicles. Biochimica Et Biophysica Acta (BBA) ‐ Molecular Basis of Disease, 1866(6), 165753. 10.1016/j.bbadis.2020.165753 32126269

[fsn32459-bib-0008] Irarrazaval, S., Allard, C., Campodonico, J., Perez, D., Strobel, P., Vasquez, L., Urquiaga, I., Echeverría, G., & Leighton, F. (2017). Oxidative stress in acute hypobaric hypoxia. High Altitude Medicine and Biology, 18(2), 128–134. 10.1089/ham.2016.0119 28326844

[fsn32459-bib-0009] Ji, Y., Gao, Y., Chen, H., Yin, Y., & Zhang, W. (2019). Indole‐3‐acetic acid alleviates nonalcoholic fatty liver disease in mice via attenuation of hepatic lipogenesis, and oxidative and inflammatory stress. Nutrients, 11(9), 2062. 10.3390/nu11092062 PMC6769627 31484323

[fsn32459-bib-0010] Kang, H. H., Kim, I. K., Lee, H. I., Joo, H., Lim, J. U., Lee, J., Lee, S. H., & Moon, H. S. (2017). Chronic intermittent hypoxia induces liver fibrosis in mice with diet‐induced obesity via TLR4/MyD88/MAPK/NF‐kB signaling pathways. Biochemical and Biophysical Research Communications, 490(2), 349–355. 10.1016/j.bbrc.2017.06.047 28623125

[fsn32459-bib-0011] Lee, S. E., Koh, H., Joo, D. J., Nedumaran, B., Jeon, H. J., Park, C. S., Harris, R. A., & Kim, Y. D. (2020). Induction of SIRT1 by melatonin improves alcohol‐mediated oxidative liver injury by disrupting the CRBN‐YY1‐CYP2E1 signaling pathway. Journal of Pineal Research, 68(3), e12638. 10.1111/jpi.12638 32053237

[fsn32459-bib-0012] Li, M., Zhang, X., Wang, B., Xu, X., Wu, X., Guo, M., & Wang, F. (2018). Effect of JAK2/STAT3 signaling pathway on liver injury associated with severe acute pancreatitis in rats. Experimental and Therapeutic Medicine, 16(3), 2013–2021. 10.3892/etm.2018.6433 30186433 PMC6122147

[fsn32459-bib-0013] Li, N., Li, Q., Bai, J., Chen, K., Yang, H., Wang, W., Fan, F., Zhang, Y., Meng, X., Kuang, T., & Fan, G. (2020). The multiple organs insult and compensation mechanism in mice exposed to hypobaric hypoxia. Cell Stress and Chaperones, 25(5), 779–791. 10.1007/s12192-020-01117-w 32430880 PMC7479670

[fsn32459-bib-0014] Li, S., Tan, H. Y., Wang, N., Zhang, Z. J., Lao, L., Wong, C. W., & Feng, Y. (2015). The role of oxidative stress and antioxidants in liver diseases. International Journal of Molecular Sciences, 16(11), 26087–26124. 10.3390/ijms161125942 26540040 PMC4661801

[fsn32459-bib-0015] Li, Y., Wu, J., Shi, R., Li, N., Xu, Z., & Sun, M. (2017). Antioxidative effects of Rhodiola Genus: Phytochemistry and pharmacological mechanisms against the diseases. Current Topics in Medicinal Chemistry, 17(15), 1692–1708. 10.2174/1568026617666161116141334 27848900

[fsn32459-bib-0016] Lin, S. Y., Dan, X., Du, X. X., Ran, C. L., Lu, X., Ren, S. J., Tang, Z.‐T., Yin, L.‐Z., He, C.‐L., Yuan, Z.‐X., Fu, H.‐L., Zhao, X.‐L., & Shu, G. (2019). Protective effects of salidroside against carbon tetrachloride (CCl4)‐Induced liver injury by initiating mitochondria to resist oxidative stress in mice. International Journal of Molecular Sciences, 20(13), 3187. 10.3390/ijms20133187 PMC6651463 31261843

[fsn32459-bib-0017] Liu, B., Wei, H., Lan, M., Jia, N., Liu, J., & Zhang, M. (2020). MicroRNA‐21 mediates the protective effects of salidroside against hypoxia/reoxygenation‐induced myocardial oxidative stress and inflammatory response. Experimental and Therapeutic Medicine, 19(3), 1655–1664. 10.3892/etm.2020.8421 32104217 PMC7027140

[fsn32459-bib-0018] Luks, A. M., & Swenson, E. R. (2015). Evaluating the risks of high altitude travel in chronic liver disease patients. High Altitude Medicine and Biology, 16(2), 80–88. 10.1089/ham.2014.1122 25844541

[fsn32459-bib-0019] McGarry, T., Biniecka, M., Veale, D. J., & Fearon, U. (2018). Hypoxia, oxidative stress and inflammation. Free Radical Biology and Medicine, 125, 15–24. 10.1016/j.freeradbiomed.2018.03.042 29601945

[fsn32459-bib-0020] Musso, G., Cassader, M., & Gambino, R. (2016). Non‐alcoholic steatohepatitis: Emerging molecular targets and therapeutic strategies. Nature Reviews Drug Discovery, 15(4), 249–274. 10.1038/nrd.2015.3 26794269

[fsn32459-bib-0021] Nath, B., & Szabo, G. (2012). Hypoxia and hypoxia inducible factors: Diverse roles in liver diseases. Hepatology (Baltimore, MD), 55(2), 622–633. 10.1002/hep.25497 PMC3417333 22120903

[fsn32459-bib-0022] Nguyen, T. L. L., Huynh, D. T. N., Jin, Y., Jeon, H., & Heo, K. S. (2021). Protective effects of ginsenoside‐Rg2 and ‐Rh1 on liver function through inhibiting TAK1 and STAT3‐mediated inflammatory activity and Nrf2/ARE‐mediated antioxidant signaling pathway. Archives of Pharmacal Research, 44(2), 241–252. 10.1007/s12272-020-01304-4 33537886

[fsn32459-bib-0023] Prieto, I., & Monsalve, M. (2017). ROS homeostasis, a key determinant in liver ischemic‐preconditioning. Redox Biology, 12, 1020–1025. 10.1016/j.redox.2017.04.036 28511345 PMC5430574

[fsn32459-bib-0024] Pu, W. L., Zhang, M. Y., Bai, R. Y., Sun, L. K., Li, W. H., Yu, Y. L., Zhang, Y., Song, L., Wang, Z.‐X., Peng, Y.‐F., Shi, H., Zhou, K., & Li, T. X. (2020). Anti‐inflammatory effects of Rhodiola rosea L.: A review. Biomedicine and Pharmacotherapy, 121, 109552. 10.1016/j.biopha.2019.109552 31715370

[fsn32459-bib-0025] Qi, Z., Qi, S., Ling, L., Lv, J., & Feng, Z. (2016). Salidroside attenuates inflammatory response via suppressing JAK2‐STAT3 pathway activation and preventing STAT3 transfer into nucleus. International Immunopharmacology, 35, 265–271. 10.1016/j.intimp.2016.04.004 27085677

[fsn32459-bib-0026] Song, T. T., Bi, Y. H., Gao, Y. Q., Huang, R., Hao, K., Xu, G., Tang, J.‐W., Ma, Z.‐Q., Kong, F.‐P., Coote, J. H., Chen, X.‐Q., & Du, J. Z. (2016). Systemic pro‐inflammatory response facilitates the development of cerebral edema during short hypoxia. Journal of Neuroinflammation, 13(1), 63. 10.1186/s12974-016-0528-4 26968975 PMC4788817

[fsn32459-bib-0027] Sun, K. H., Yu, C. L., Tang, S. J., & Sun, G. H. (2000). Monoclonal anti‐double‐stranded DNA autoantibody stimulates the expression and release of IL‐1beta, IL‐6, IL‐8, IL‐10 and TNF‐alpha from normal human mononuclear cells involving in the lupus pathogenesis. Immunology, 99(3), 352–360. 10.1046/j.1365-2567.2000.00970.x 10712664 PMC2327177

[fsn32459-bib-0028] Sundaram, S. S., Halbower, A., Pan, Z., Robbins, K., Capocelli, K. E., Klawitter, J., Shearn, C. T., & Sokol, R. J. (2016). Nocturnal hypoxia‐induced oxidative stress promotes progression of pediatric non‐alcoholic fatty liver disease. Journal of Hepatology, 65(3), 560–569. 10.1016/j.jhep.2016.04.010 27501738 PMC4992457

[fsn32459-bib-0029] Wang, C., Wang, R., Xie, H., Sun, Y., Tao, R., Liu, W., Li, W., Lu, H., & Jia, Z. (2016). Effect of acetazolamide on cytokines in rats exposed to high altitude. Cytokine, 83, 110–117. 10.1016/j.cyto.2016.04.003 27104804

[fsn32459-bib-0030] Xiao, M. H., Xia, J. Y., Wang, Z. L., Hu, W. X., Fan, Y. L., Jia, D. Y., Li, J., Jing, P. W., Wang, L., & Wang, Y. P. (2018). Ginsenoside Rg1 attenuates liver injury induced by D‐galactose in mice. Experimental and Therapeutic Medicine, 16(5), 4100–4106. 10.3892/etm.2018.6727 30402153 PMC6200997

[fsn32459-bib-0031] Xiong, Y., Wang, Y., Xiong, Y., Gao, W., & Teng, L. (2020). Salidroside alleviated hypoxia‐induced liver injury by inhibiting endoplasmic reticulum stress‐mediated apoptosis via IRE1alpha/JNK pathway. Biochemical and Biophysical Research Communications, 529(2), 335–340. 10.1016/j.bbrc.2020.06.036 32703432

[fsn32459-bib-0032] Yang, Y. Q., Yan, X. T., Wang, K., Tian, R. M., Lu, Z. Y., Wu, L. L., Xu H.‐T., Wu Y.‐S., Liu X.‐S., Mao W., Xu P., & Liu, B. (2018). Triptriolide alleviates lipopolysaccharide‐induced liver injury by Nrf2 and NF‐kappaB signaling pathways. Frontiers in Pharmacology, 9, 999. 10.3389/fphar.2018.00999 30210350 PMC6124152

[fsn32459-bib-0033] Yang, Z. R., Wang, H. F., Zuo, T. C., Guan, L. L., & Dai, N. (2016). Salidroside alleviates oxidative stress in the liver with non‐ alcoholic steatohepatitis in rats. BMC Pharmacology and Toxicology, 17, 16. 10.1186/s40360-016-0059-8 27075663 PMC4831194

[fsn32459-bib-0034] Yuan, Y., Wu, X., Zhang, X., Hong, Y., & Yan, H. (2019). Ameliorative effect of salidroside from Rhodiola Rosea L. on the gut microbiota subject to furan‐induced liver injury in a mouse model. Food and Chemical Toxicology, 125, 333–340. 10.1016/j.fct.2019.01.007 30654097

[fsn32459-bib-0035] Zhang, L., Wang, H., Fan, Y., Gao, Y., Li, X., Hu, Z., Ding, K., Wang, Y., & Wang, X. (2017). Fucoxanthin provides neuroprotection in models of traumatic brain injury via the Nrf2‐ARE and Nrf2‐autophagy pathways. Scientific Reports, 7, 46763. 10.1038/srep46763 28429775 PMC5399453

[fsn32459-bib-0036] Zheng, K., Sheng, Z., Li, Y., & Lu, H. (2014). Salidroside inhibits oxygen glucose deprivation (OGD)/re‐oxygenation‐induced H9c2 cell necrosis through activating of Akt‐Nrf2 signaling. Biochemical and Biophysical Research Communications, 451(1), 79–85. 10.1016/j.bbrc.2014.07.072 25063033

[fsn32459-bib-0037] Zheng, T., Yang, X., Li, W., Wang, Q., Chen, L., Wu, D., Bian, F., Xing, S., & Jin, S. (2018). Salidroside attenuates high‐fat diet‐induced nonalcoholic fatty liver disease via AMPK‐dependent TXNIP/NLRP3 pathway. Oxidative Medicine and Cellular Longevity, 2018, 8597897. 10.1155/2018/8597897 30140371 PMC6081551

[fsn32459-bib-0038] Zhu, Y., Zhang, Y. J., Liu, W. W., Shi, A. W., & Gu, N. (2016). Salidroside suppresses HUVECs cell injury induced by oxidative stress through activating the Nrf2 signaling pathway. Molecules (Basel, Switzerland), 21(8), 1033. 10.3390/molecules21081033 PMC6273208 27517893

